# L'ostéogenèse imparfaite: à propos d'un cas

**DOI:** 10.11604/pamj.2015.21.83.6416

**Published:** 2015-06-03

**Authors:** Najeh Hsayaoui, Chaouki Mbarki, Saoussen Melliti, Youssef El Cadhi, Fatma Douik, Sana Mezghanni, Hedhili Oueslati

**Affiliations:** 1 Service de Gynécologie Obstétrique Hôpital Ben Arous, Tunis, Tunusie; 2 Service de Radiologie Hôpital Ben Arous, Tunis, Tunusie

**Keywords:** Ostéogenèse imparfaite, échographie, diagnostic, Osteogenesis imperfecta, echography, diagnosis

## Abstract

L'ostéogenèse imparfaite est une maladie héréditaire caractérisée par une fragilité osseuse secondaire à un défaut de synthèse du collagène de type I. Le diagnostic est suspecté devant des signes échographiques évocateurs et confirmé par une étude génétique. Nous rapportons un cas d'ostéogenèse imparfaite de découverte tardive au troisième trimestre chez une patiente qui n'a pas suivi sa grossesse.

## Introduction

L'ostéogenèse imparfaite (OI) est une maladie héréditaire caractérisée par une fragilité osseuse secondaire à un défaut de synthèse du collagène de type I [1]. Le tableau clinique est variable pouvant inclure des fractures prénatales et décès périnatal, ou des formes très frustes. Des troubles extrasquelettiques peuvent être associés à des degrés variables. C'est une maladie rare qui touche hommes et femmes, sans prédominance ethnique ou raciale [2].

## Patient et observation

**Figure 1:**
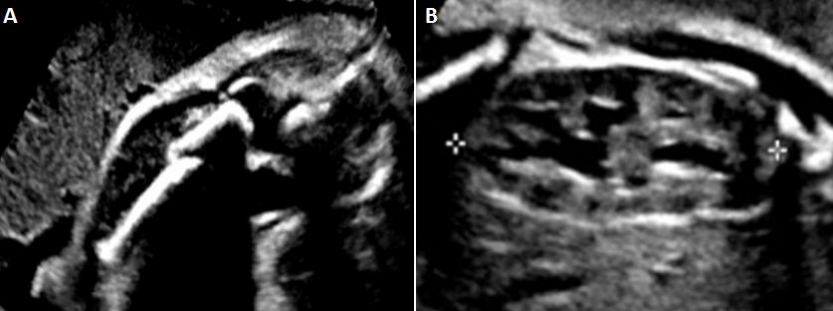
Image échographique de l'ostéogenèse imparfaite; (A) fracture déplacée du fémur, (B) aspect allongé du crane

**Figure 2:**
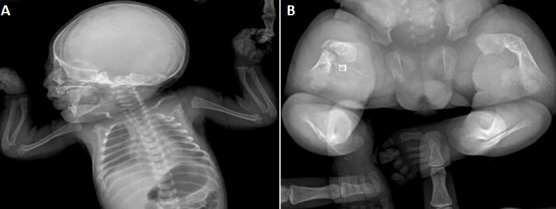
Radiographie de squelette après la naissance; (A) défaut d'ossification du crâne, membres supérieures très courts, côtes déformées porteuses de cals, (B) membres inférieures courts et incurvés

Mme H.Z est âgée de 26 ans. C'est une deuxième geste. Elle a un enfant en bon état de santé apparente. Il n'y a pas de notion de mariage consanguin. Mme H.Z. n'a pas suivi sa grossesse en cours. Elle a consulté pour la première fois à 32 SA pour échographie obstétricale. Cet examen a révélé un foetus malformé présentant un nanisme micromélique associant des membres courts et incurvés avec fracture déplacée du fémur droit, le crâne est allongé (Figure 1). Le diagnostic d'OI a été fortement évoqué. La patiente a eu une corticothérapie anténatale avec accouchement par césarienne à 38 SA. L'examen néonatal a objectivé une déformation de la cuisse droite en rapport avec la fracture pathologique survenue « in utéro ». La radiographie du squelette a montré un défaut majeur de l'ossification touchant tous les os (Figure 2) associé à des déformations des os des membres (avant-bras, cuisses jambes). L'enfant a survécu pendant une année. Au cours de cette période, il a eu des multiples fractures des membres supérieures et inférieures et il est décédé à l’âge de un an dans un tableau de détresse respiratoire.

## Discussion

L'ostéogenèse imparfaite fait partie d'un groupe d'affections héréditaires à transmission dominante, caractérisée par une anomalie de la synthèse du collagène de type 1 (production réduite et collagène anormal). C'est une maladie rare. Elle atteint un nouveau-né sur 25 à 50.000, sans prédominance de sexe ou de race et sans distribution géographique préférentielle [1]. Les fractures multiples par fragilité osseuse représentent la principale manifestation de la maladie, et sont responsables de la morbidité et la mortalité de l'ostéogenèse imparfaite par insuffisance respiratoire secondaire aux fractures costales [3]. Des troubles extra squelettiques sont associés à des degrés variables avec retentissement plus ou moins important. Une laxité ligamentaire qui peut compromettre la stabilité articulaire, des troubles oculaires et auditives, des anomalies cardio-vasculaires, des troubles de l'hémostase, des affections rénales et une dentinogenèse imparfaite sont les manifestations les plus fréquentes [1, 2].

La classification de Sillence et al [4], complétée en 2004 par Rauch et Glorieux [5], classiquement la plus employée, distingue actuellement sept formes. En dehors des formes de sévérité extrême, il peut être difficile de classer un patient lors de la découverte de la pathologie, surtout dans les premières années de vie. Il peut donc être utile d'utiliser une classification selon l’âge d'apparition [6].

Le diagnostic anténatal est évoqué généralement devant des anomalies osseuses à type de fracture, incurvation, déformation ou raccourcissement. Rarement une déformabilité du crâne sous le passage de la sonde d’échographie peut attirer l'attention. Le pronostic est difficile à établir précocement. L’évolution est imprévisible. Des fractures multiples et des déformations osseuses peuvent se voir avec retentissement statural et respiratoire variable [1]. Le diagnostic de l'OI dans ses formes à révélation postnatale est clinique, reposant sur les manifestations squelettiques décrites ci-dessus ainsi que les manifestations extra squelettiques. Les différentes manifestations extra squelettiques sont variables. On peut noter une laxité ligamentaire avec retentissement variable sur la statique, une coloration bleutée des sclérotiques due à la transparence excessive de la sclérotique, une perte de l'audition, une atteinte du système cardiovasculaire. Les ecchymoses et les épistaxis sont fréquentes chez l'enfant atteint d'ostéogenèse imparfaite ainsi que les atteintes rénales neurologiques et hépatiques [2].

Bien que le diagnostic de l'OI est basé sur la clinique et l’échographie, les progrès de la génétique moléculaire ont permis de localiser les gènes responsables de la maladie. Il s'agit de deux gènes, Col 1 A1 situé sur le bras long q du chromosome 17 entre les positions 21,31 et 22,15, et Col 1 A2 situé sur le bras long q du chromosome 7 entre les positions 21,3 et 22, 1. Ces deux gènes Col 1 A1 et Col1 A2 codent respectivement pour la synthèse des chaînes a1 et a2 du collagène de type I. Ces mutations sont pour la plupart dominantes, elles sont soit de novo, ce qui est le plus fréquent, soit résultent d'une mosaïque d'un des parents [7, 8].

Le traitement de cette affection est symptomatique. Le pronostic fonctionnel dépend de la sévérité de l'atteinte et de sa prise en charge. L'utilisation récente des bisphosphonates, associée à la stimulation motrice et à la chirurgie, a beaucoup amélioré l'autonomie des sujets ayant une forme grave. Le pronostic vital est lié à l'atteinte respiratoire corrélée à la sévérité des déformations rachidiennes [2].

## Conclusion

Le diagnostic d'une ostéogenèse imparfaite doit etre évoqué échographiquement devant des déformations des membres. Un conseil génétique doit être proposé en cas d'antécédent d'une ostéogenèse imparfaite dans la famille.
